# A structure-based Multiple-Instance Learning approach to predicting *in vitro *transcription factor-DNA interaction

**DOI:** 10.1186/1471-2164-16-S4-S3

**Published:** 2015-04-21

**Authors:** Zhen Gao, Jianhua Ruan

**Affiliations:** 1 Department of Computer Science, The University of Texas at San Antonio, One UTSA Circle, San Antonio, TX 78249, USA

## Abstract

**Background:**

Understanding the mechanism of transcriptional regulation remains an inspiring stage of molecular biology. Recently, *in vitro *protein-binding microarray experiments have greatly improved the understanding of transcription factor-DNA interaction. We present a method - MIL3D - which predicts *in vitro *transcription factor binding by multiple-instance learning with structural properties of DNA.

**Results:**

Evaluation on *in vitro *data of twenty mouse transcription factors shows that our method outperforms a method based on simple-instance learning with DNA structural properties, and the widely used *k*-mer counting method, for nineteen out of twenty of the transcription factors. Our analysis showed that the MIL3D approach can utilize subtle structural similarities when a strong sequence consensus is not available.

**Conclusion:**

Combining multiple-instance learning and structural properties of DNA has promising potential for studying biological regulatory networks.

## Introduction

Modeling of transcription factor binding sites (TFBS), sometimes also referred to as transcription factor binding motifs, is a crucial step towards understanding molecular regulatory networks. Among the popular methods for modeling TFBS, position-specific weight matrix (PWM) [1,2] and *k*-mer based approaches [3,4] have gained great success [5,6]. Providing a probability score for each of the four nucleotide bases of each position of a TFBS, PWM-based approaches are intuitive for representing the sequence preferences of a transcription factor (TF), and easy to visualize for the TFBS models. However, these approaches have some limitations, including inefficiency in optimizing PWM, difficulty to represent some TFBSs (such as structural motifs and those with variable lengths) by a single PWM, and incapability to capture positional dependency [3,4,7,8]. In contrast, *k*-mer based approaches use a word-based sequence of length *k *to enumerate possible instances of a motif. Compared to PWM-based approaches, the *k*-mer based approaches can be designed to make fewer assumptions about the degree of TFBS degeneracy, length of the binding sites, and position dependences of a motif. The *k*-mer based approaches have been found to provide more accurate models than PWMs in general [5,6,9]. But these models usually involve too many parameters and may not unravel the underlying knowledge of TF-DNA interactions.

A TF binding site is usually 8 to 12-mer base pair, determined by the sequence specificity and its physicochemical properties (structural and chemical properties), which are overlooked by both the PWM based and the *k*-mer based approaches. In [10], a novel TFBS modeling and predicting approach is presented, where the sequence-specific chemical and structural features of DNA are applied. Based on their evaluation using an *in vivo *ChIP-chip dataset, their method outperforms four previous methods [1,11-13] by reporting fewer false positive matches. Their method provides a new perspective for understanding TF-DNA interactions. On the other hand, the *in vivo *protein-DNA interactions observed in ChIP-chip assays are not necessarily direct [14], as some TFs tend to interact with DNA extensively through other partners. Therefore, an evaluation on a proper *in vitro *dataset would be more appropriate to reveal the benefit of such physicochemical features in modeling TF-DNA interactions.

Protein-binding microarray (PBM) is a high-throughput experiment used to measure the *in vitro *binding affinity of a given TF to the sequences on the probe array [15]. A typical design of the array consists of an exhaustive enumeration of all possible 10-mers concatenated into ∼40,000 unique probe nucleic acid sequences, each containing 35 bases. The PBM score represents the relative binding affinity of a given TF to each probe sequence on the array. Because typical confounding factors such as transcription cofactors present in ChIP-based experiments are eliminated, PBM data provides an excellent information source to develop physicochemical models for TF-DNA interactions. On the other hand, the currently available physicochemical features are mainly 3-mer or 4-mer based [10]. Direct mapping of the 3-mer or 4-mer based meta-features to the candidate DNA binding sequences as in the work of [10] may not reflect the TF-DNA binding nature, since a TFBS usually spans 6 to 12 base pairs (bp), and its exact location within the PBM probe sequence is unknown. As a result, conventional machine learning algorithms, which rely on well-structured instance (feature vector) and label pairs, may not work well in modeling PBM data.

In this paper we propose a novel approach, MIL3D, to predict *in vitro *transcription factor binding based on the structural properties of DNA using the so-called multiple-instance learning (MIL), which was originally developed in the mid '90s to deal with uncertainty in instance labels and has found many interesting applications in bioinformatics and information retrieval [16-19]. In a conventional classification problem, the input is a set of instances (feature vectors), each of which is labeled positive or negative. In contrast, the input of MIL is a set of bags that can have many instances, but the instances are not individually labeled - instead, the labels are associated with bags. The common assumption is that a bag with a positive label contains at least one positive instance (whose identity is unknown), while all instances in the negative bag should have negative labels. The MIL framework fits the TFBS modeling scenario very well, because it is commonly assumed that a DNA sequence that can be bound by a TF (positive sequence) should contain one or more TFBSs, while a DNA sequence that cannot be bound by the TF (negative sequence) should has no TFBS. The exact location of the TFBS within the positive sequence is typically not fixed, although some preference might exist. Therefore, it is fairly intuitive to consider each DNA sequence as a bag, and any subsequence that can be a potential binding site as an instance.

In our algorithm, we treat each PBM probe sequence as a bag, and label the bags positive or negative according to the affinity level from the TF binding data. Each PBM probe is then decomposed into a set of *k*-mers using a simple sliding-window approach, and each *k*-mer is represented by a vector describing its structural properties. We then apply an existing MIL algorithm to learn a classification model that can correct predict the labels for the bags.

To demonstrate the advantage of our approach, we compared the performance of our algorithm with the conventional single-instance based learning (SIL), and with the simple *k*-mer counting method. Experimental results on PBM data of twenty mouse TFs showed that our method outperformed those methods with significant margins. On the other hand, it is also worth noting that our goal in this current paper is not a state-of-the-art method to predict TF-DNA interaction with the highest accuracy. To our knowledge, this is the first work that demonstrates the feasibility of using the MIL paradigm and structural properties in modeling TF-DNA interactions. We believe that many of the existing *k*-mer based methods for predicting TF-DNA interactions, which often involve filtering, normalization, and transformation of the binding data, can be combined with the key idea proposed in this paper to obtain a more accurate model.

## Materials and methods

### The *in vitro *transcription factor-DNA binding data

The protein binding microarray (PBM) data is acquired from [5]. Two completely different array designs, each of which consists of 40,000 unique 35-mers, are used for twenty different mouse TFs. In the arrays, all possible 10-mers, and 32 copies of every non-palindromic 8-mer are included, presenting an unbiased study of TF binding preferences. The data were used for training in the Dialogue on Reverse-Engineering Assessment and Methods (DREAM) competition [20] and the data is freely available on the DREAM5 competition website upon registration. In our experiment, we selected 3000 probes with the highest binding signals as positive sequences and 3000 probes with the lowest binding signals as negative for each of the twenty TFs. We used the two different sets of arrays for training and testing respectively. As the training and testing data are completely separate, and actually come from different sources, this setting makes the prediction problem more challenging and the results less likely to be influenced by biases, compared to evaluations based on, for example, 10-fold cross-validation, where training and testing data could share some unknown similarities and bias the evaluation results.

### MIL model for TF-DNA binding

**Figure 1:**
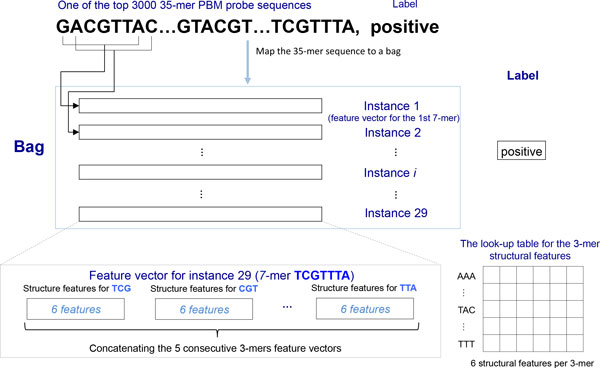
**Mapping structural features to DNA sequences by MIL3D**. This figure illustrates how to map the meta-features to a 35-mer DNA string by our MIL3D method, assuming that there is only one meta-feature table - STRUC-3-mer and the *k*-mer length is 7.

Conventional classification algorithms deal with instances that consist of feature vector-label pairs, where each instance (e.g., probe sequence) is represented by a well-structured feature vector and has a label. The modeling task is to extract useful information (e.g., a subset of features) to map to the labels. A crucial difference between MIL and conventional learning algorithms is that in MIL, labels are associated with bags (as opposed to instances), and each bag can contain multiple instances. While the instances in the same bag do not have their own labels, depending on the actual MIL algorithm implementation, it is assumed that they will contribute to the label of the bag in a binary or probabilistic way. In modeling PBM data, it is reasonable to assume that a probe sequence with high binding affinity (positive sequence) contains one or more binding sites, while a probe sequence with low affinity (negative sequence) does not have any binding sites. Therefore, we consider each candidate binding site (*k*-mer, *k *∈ [5, 8] in this study) as an instance and all possible *k*-mers in a probe sequence as a bag. Bags are labeled positive or negative according to the binding affinity of the probe sequences as mentioned above. Each instance is mapped to a single feature vector representing the structural properties of the *k*-mer, as will be discussed later (Figure 1).

To apply MIL to model the transcription factor binding site, we use the multiple-instance-wrapper algorithm [21] implemented in the WEKA machine learning package [22], and used the popular C4.5 decision tree algorithm (named J48 in WEKA) as the base single-instance classifier. The choice of C4.5 is because it is a simple and less biased classifier - it does not need to adjust a parameter to optimize its performance, and the number of features of an instance has little influence on its performance. Moreover, the difference of range and scale among each feature has little influence on the performance as well.

The wrapper-based MIL approach enables us to compare MIL-based TF-DNA interaction models and single-instance based model in a relatively unbiased manner, because the wrapper-based method can use any traditional single-instance classifiers as a base classifier. In contrast, a non-wrapper based MIL algorithm does not use SIL classifiers directly, which makes it hard to underpin the source of the performance difference between the two. The wrapped approach is derived from the assumption that a bag's label is formed by the overall contribution of all the instances within the bag, which is also consistent with the understanding that multiple binding sites may contribute to recruiting TF additively. Based on the above assumption, at the learning time, the wrapper algorithm converts the MIL training and testing data to single-instance based learning data by giving each instance a label that is the same as its bag, and each instance is initially assigned a weight proportional to the inverse of the size of the bag that they belong to. In our case, as every bag has the same size (35−*k*+1 instances each representing a *k*-mer), the initial weight for every instance is $\frac{1}{35-k+1}$. This setting is because we do not have any prior knowledge for the location of the TFBS within the probe sequence, and therefore we assume that all *k*-mers within the 35-mer have the same probability being the real binding site, which will be updated iteratively when the model becomes more accurate. At prediction time, the class probability for a bag is estimated by averaging the class probabilities assigned to the individual instances in the bag. Moreover, the class probability for each individual instance can also be extracted from the positive bags to identify possible binding site(s) for each probe sequence. As the result of the above designs, different from most of the other non-wrapper based MIL classifiers, the wrapper algorithm can be combined with any traditional single-instance classifiers, which enables an unbiased comparison between our MIL-based TFBS modeling method with the traditional SIL based TFBS modeling method.

The choice of decision tree rather than other popular algorithms such as support vector machines (SVM) is because of the relative parameter-free robust performance and the implicit feature selection provided by the former method, which makes it ideal for evaluating and comparing the performance of the MIL-based and SIL-based approaches. For example, the performance of SVM approach is very sensitive to kernel choices and other parameters including the soft margin parameter and feature normalization method. As the *k*-mer based approaches and our structure-based MIL approach have very different number of features and different feature characteristics, (e.g., in *k*-mer based approach, all features are small integers, while in our method the features are typically real values and different features can have different distributions), we feel a simple base classifier such as a decision tree can provide a more fair comparison.

### The meta-features of DNA structural properties

The structural properties of DNA is obtained from [10], which describe the steric and conformational rigidity properties of DNA. The structural features of DNA oligomers were defined based on the predicted average 3-dimensional structures of short DNA sequences, which were determined via MD simulations [23]. The structural feature table of 3-mer is a 64 by 6 matrix, which contains six real value "base" parameters for each middle base pair of all the 64 (4^3^) different 3-mers. The base parameters measure the relative relations between two fundamental units of the middle based pair of a 3-mer in a 3-dimensional space. The six base parameters are shear, buckle, stretch, propeller, stagger and opening [24].

### Mapping PBM sequences to feature vectors

Figure 1 shows the steps of mapping the 35-mer PBM probe sequence to the structural feature vectors by our MIL3D method. In this example the *k*-mer length is specified as 7. As mentioned before, each probe sequence is treated as a bag and has a negative or positive label depending on its binding affinity to a particular TF. Each of the 29 shifted 7-mers in the 35-mer is treated as an instance, which does not have a label itself. For each instance (7-mer), we obtain the structural features of each of the 5 triplets using the structural feature table mentioned above as a lookup table. The feature vectors for the 5 triplets from the same 7-mer are then concatenated, in the same order as they appear in the 7-mer, to form a single feature vector for the 7-mer, which is an instance in the MIL model.

Table 1 shows the detailed profiles of the MIL3D method as well as the comparison approaches, including a SIL model with the structural meta-features - SIL3D [10], and the simple traditional *k*-mer based approaches. In SIL3D, each 35-mer is treated as an instance, whose feature vector is simply the concatenation of the structure features of the 33 continuous 3-mers from the 35-mer, and SIL instead of MIL is applied to learn the classification model. The *k*-mer based approach also uses SIL. It counts the number of occurrences for every possible *k*-mer and uses the *k*-mer counts as the feature vector in the model. We have tried *k*-mer length from 5 to 8 for the MIL3D method and *k*-mer length from 3 to 8 for the simple counting method. In addition, for the *k*-mer based method, we included a '3+4+5mer counting' model, which uses counts of all 3-, 4-and 5-mers as features. The comparison between MIL3D and SIL3D will show the benefit of using MIL, while the comparison between MIL3D and the *k*-mer based methods is expected to show the benefit from using the structural features and MIL.

**Table 1:** Summary of different feature models.

Name	Length of *k*-mer kernel	Number of instances per bag	Number of features per instance	Description
MIL3D_*k*mer	*k *∈ [5, 8]	35-*k*+1	*k*-3+1 triplets per *k*-mer * 6 base structural features per triplet	For each of the 35-*k*+1 different continuous *k*-mers in the 35-mer, for each of the *k*-3+1 triplets, map the structural features to the *k*-mer sequentially. The feature vector of one *k*-mer represent an instance, and the 35-*k*+1 instances form a bag.

SIL3D	3	1	198 (6 structural features per triplet * 33 continous 3-mers in the 35-mer)	For each of the 33 different continous 3-mers in the 35-mer, map the 6 structural features to the 3-mer.

*k*mer_counting	*k *∈ [3, 8]	1	4*^k ^*(number of occurrences of all different *k*-mers)	For each 35-mer DNA sequence in the PBM array, count the number of occurrences for each of the 4*^k ^k*-mers; map the *k*-mer counter table to the PBM sequence. This *k*-mer based method has been widely used in the previous decades and has been proven to be still very effective at present [5].

3+4+5mer_counting	3, 4 and 5	1	1344 (64+256+1024)	For each 35-mer DNA sequence in the PBM array, map the above 3 counter tables (including 3-mer, 4-mer and 5-mer tables) to the sequence.

## Results

**Figure 2:**
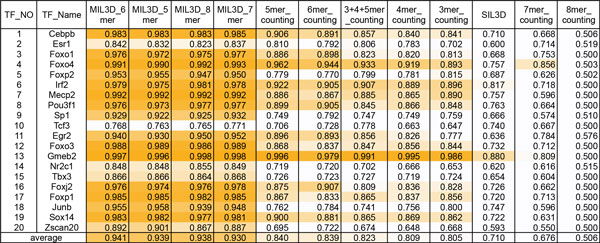
**Comparison of algorithm performance on PBM data**. The color scale indicates the AUC score - the darker the color, the higher the AUC score. For each model shown in each column, the AUC scores of the predicted results for twenty TFs are given.

The predicting Area Under ROC (AUC) scores for each model-TF pair are given in Figure 2. As shown, SIL3D consistently has the worst performance, which is as expected. This is because in SIL3D, the 3-mer based feature vectors are concatenated by the order they appear in the 35-mer, and therefore bears the implicit assumption that the same position different probe sequences have the same contribution in determining TF binding affinity, which is in contrary to the common knowledge that the TF-DNA interaction is relatively independent of the position of the binding site. In contrast, MIL3D does not explicitly consider the position of the 3-mers in the probe sequence, but instead attempts to find the most contributing instance in a bag. In other words, MIL helps find the real TFBSs within a PBM probe sequence.

The MIL3D method performs significantly better than the traditional *k*-mer counting method. Furthermore, changing of instance (*k*-mer) length in the MIL3D model has almost no impact on the performance while the performance of *k*-mer based method significantly depends on the value of *k*. The best performance of the *k*-mer based method is with *k *equal to 5. Larger values of *k *cause a significant degradation of the performance of the *k*-mer based method, presumably due to overfitting associated with the exponentially growing number of features and sparsity of the feature vectors when *k *increases. For example, each instance of the 7-mer counting has 16384 features while the total number of instances in our experiment is only 6000. In contrast, the number of features in MIL3D is linear to the number of 3-mers contained in the *k*-mer, and therefore is relatively stable when the value of *k *changes. While the actual TFBS length might be different from *k*, the MIL3D model can overcome the problem by modeling the TFBS with multiple overlapping instances, each of which can be a partial binding site.

As shown in Figure 2, MIL3D models are consistently better than *k*-mer based models for almost all TFs, often by a significant margin, except for Tcf3, where the 3+4+5-mer counting approach works slightly better than MIL3D models. The average AUC for MIL3D (0.94) is significantly higher than the average AUC for the 5mer counting method (0.84) (*p*-value = 1e-7, paired t-test). Among all MIL3D models, there are only five TFs out of twenty with AUCs below 0.91, while for the 5-mer counting models, there are only 5 TFs with AUCs above 0.90. With the MIL3D method, there are 11 TFs with AUCs above 0.95, while with the 5-mer counting method, only has 2 TFs (Foxo4 and Gmeb2) have AUCs greater than 0.95. The performance of MIL3D on Esr1, Tcf3, Nr2C1 and Tbx3 are low compared to other TFs (AUC<0.86), despite being considerably better than the 5-mer counting method, marking possible areas for improvement.

**Figure 3:**
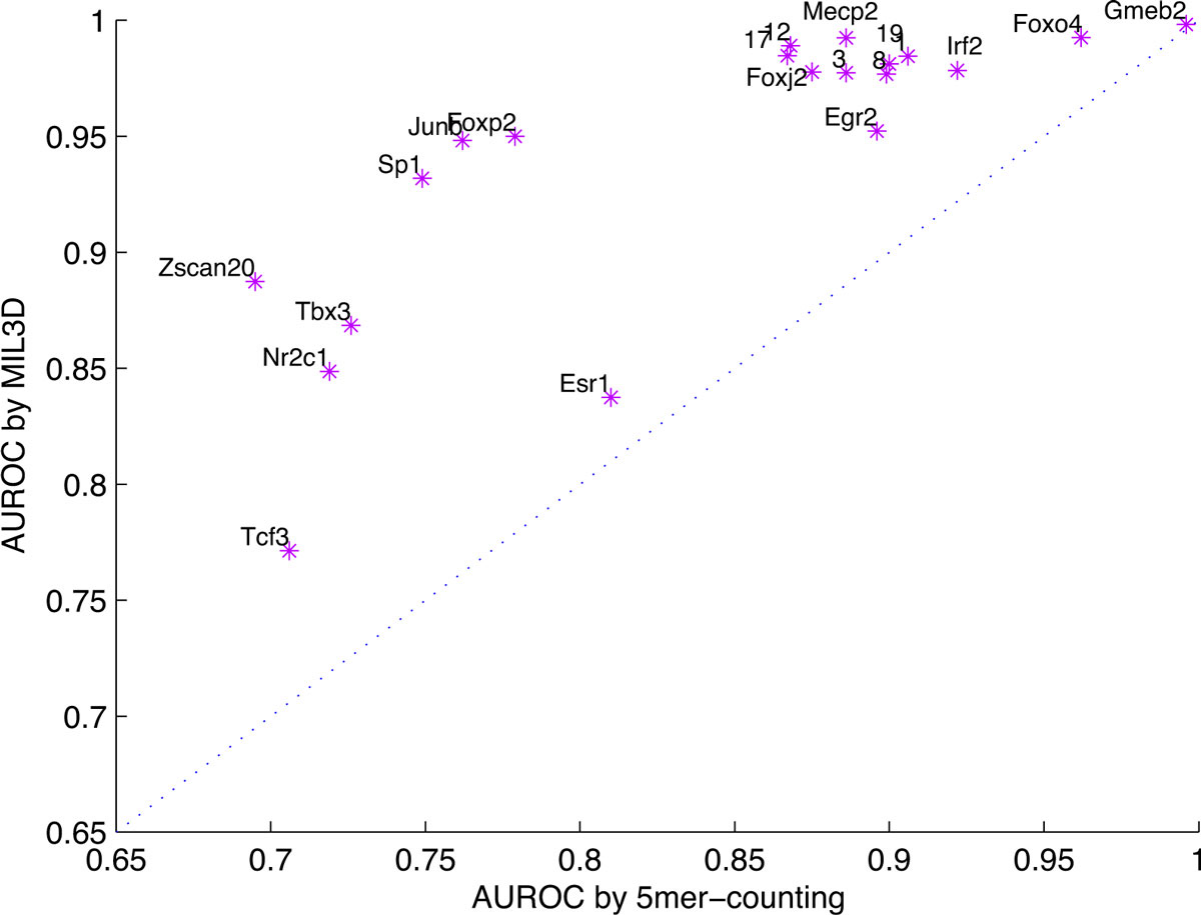
**Scatter plot for performance comparison between 5mer-counting method and MIL3D**. The twenty purple stars represent the twenty TFs. The names of the TFs are shown on the upper left corner of each of the corresponding star signs, except for TF 1, 3, 8, 12, 17 and 19. They are shown by numbers to avoid text overlapping. TF 1, 3, 8, 12, 17 and 19 represent Cebpb, Foxo1, Pou3f1, Foxo3, Foxp1 and Sox14 respectively.

Figure 3 compares the performance of MIL3D 7-mer and 5-mer counting on every TF. In the following result discussion, we use MIL3D to represent MIL3D 7-mer for short. The reason that we choose 7 as the main length of motif model is because 7 is one of the most common lengths of a motif core. It shows a direct performance comparison between the conventional 5mer-counting method and MIL3D, based on the AUC score. We can see that for the prediction of TF with the lowest score, such as Tcf3 (0.771 by MIL3D and 0.706 by 5mer-counting), both methods do not perform well, while for some highest scoring TFs, such as Foxo4 and Gmeb2, the performance of the two methods also do not have much differences (all the AUCs for them is above 0.962). However, for the TFs with a relatively moderate predicting performance by 5mer-counting, such as Sp1, Junb and Foxp2, the performance gained by MIL3D is significant. Note that Junb binding preference is quite complex and shows non-canonical patterns, with multiple variable-length consensuses including TGA[G/C]TCA and TGACGT[C/T]A [25,26]. The AUC for Junb by 5mer-counting is 0.762, while it is 0.921 by MIL3D (+0.159 gain), which shows the advantage of MIL3D of modeling complex binding site. The AUC accuracy for Sp1 by 5mer-counting is 0.749, while by MIL3D it is 0.915 (+0.166 gain). The AUC for Foxp2 is 0.779 by 5mer-counting, while it is 0.940 by MIL3D (+ 0.161 gain).

**Figure 4:**
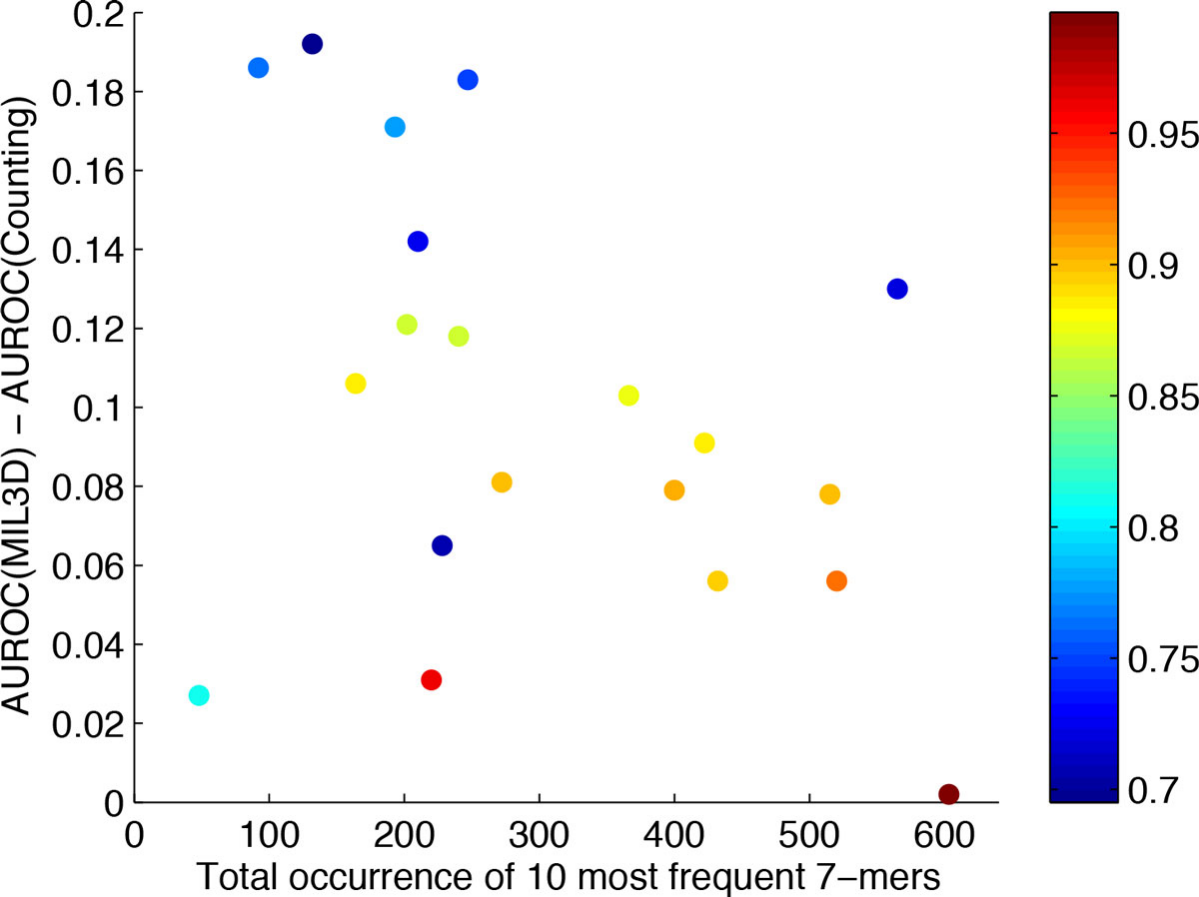
**Correlation between 7-mer frequency and performance gain by MIL3D**. Not considering TF Esr1 (the light blue, bottom left data point), for which both MIL3D and simple counting had low accuracy, a significant negative correlation (Pearson correlation coefficient = −0.61, *p*-value = 0.006) is observed between the total occurrence of the top-10 7-mers in the test sequences and the performance gain achieved by MIL3D. Color of data points represents the AUC by the simple counting method.

To further investigate the fundamental reason for the improved prediction accuracy of MIL3D, we further analyzed the putative binding sites of the TFs by MIL3D. To this end, we ranked all 7-mers in the positive test probe sequences by their predicted probability of binding, and extracted the top ten most frequent 7-mers that have a predicted binding probability at least 0.85. Table 2 shows those 7-mers and their frequency. In general, a high total occurrence of these ten 7-mers or the existence of a few dominating 7-mers indicates stronger consensus binding site. Interestingly, the TFs that the simple counting method performed well for, such as Foxo4 and Gmeb2, tend to have a strong consensus motif, while the TFs that the simple counting method failed to model do not seem to have a strong consensus motif. A significant positive correlation (Pearson correlation coefficient = 0.45, *p*-value = 0.05) exists between the AUC of 5-mer counting and the total occurrence of the top-ten 7-mers, while such correlation does not exist between the AUC of MIL3D and 7-mer occurrences (Pearson correlation coefficient = 0.27, *p*-value = 0.25). As shown in Figure 4, the performance gain of MIL3D over 5-mer counting is negatively correlated with 7-mer occurrences (Pearson correlation coefficient = −0.42, *p*-value = 0.07). The TF Esr1 seems to lack strong consensus and both MIL3D and 5mer-counting performed relatively poorly. When Esr1 is excluded, the correlation between performance gain by MIL3D over 5-mer counting and 7-mer occurrence becomes much more significant (Pearson correlation coefficient = −0.61, *p*-value = 0.006). This analysis suggests that while a simple counting method works well for TFs that contain a single dominating consensus, the MIL3D approach is more versatile and can utilize subtle structural similarities when a strong sequence consensus is not available.

**Table 2:** Occurrence of ten most frequent high-scoring 7-mers in positive probe sequences for each TF (only 7-mers with a predicted probability of binding > 0.85 are considered.)

Cebpb		Esr1		Foxo1		Foxo4		Foxp2	
GATTGCA	48	AGTCAAG	9	ACAAACA	53	GATAAAC	30	TGTATAC	32
TGTTGCA	43	ATGATCT	7	TGTTATT	52	AATAAAC	29	TGTTGTA	21
TATTGCG	42	ACGTCGA	6	TGTTTTT	47	CTATTTA	27	TGTGTAC	20
CATTGCA	39	ACGTTCT	5	TGTTTGT	44	AATAAAT	23	CTTGATA	12
CATTGCG	38	AGGTGCA	4	ACAACAT	41	CATAAAC	22	AATATCC	10
GATTGCG	35	ATGTTCT	4	TGTTGAT	41	GATAAAT	21	TGTGCTT	10
TTTTGCA	35	ACGATAT	3	TGTTATC	32	CATAAAT	19	TCTGTTC	10
CGTTGCA	33	AGTCAGC	3	ACAATAA	31	CTATTTG	11	ACTATCC	9
TTGCGAA	26	ACGCCCT	2	AAACAGG	21	GTATTTC	7	ATTATCC	9
Total	339	Total	43	Total	362	Total	189	Total	133

Irf2		Mecp2		Pou3f1		Sp1		Tcf3	

TTTCATT	58	CACACAG	24	TTAATTA	62	TCCGCCC	38	CACCTGG	60
TTTCGAT	56	ACACAGG	24	CTAATTA	60	ACCGCCC	35	CACCTGA	43
TTTCGGT	56	GACACAG	17	ATTAATT	57	TGGGCGG	30	TCAGCTG	16
TTTCGCT	54	ACACAGC	15	GTAATTA	53	GCCGCCC	26	GAATGCA	13
TTTCACT	53	TACACAG	13	ATTAATA	50	GGGCGGG	23	CACCAGG	8
TTTCAGT	46	ACACGCT	13	ATTAATG	46	TACTCCA	14	TCGTCAC	7
TTTTCGT	46	ACACGGC	13	TATAATT	43	TCTGGGC	11	CACCAGA	5
TTTCTAT	43	TAAAGTA	10	ATTATTA	42	TGGGAGG	11	GCCAGAA	3
TTTTCAT	41	CACTGAC	9	AATAATT	40	GGGAGGG	10	TCAGCTT	3
Total	453	Total	138	Total	453	Total	198	Total	158

Egr2		Foxo3		Gmeb2		Nr2c1		Tbx3	

CACCCAC	60	GCTGTTT	22	GTACGTA	68	ATGACCC	61	TAGGTGT	32
CTCCCAC	56	CTTATTT	21	TGACGTA	65	GTGACCC	60	AAGGTGT	30
GGCCCAC	47	AATATTT	20	GACGTAA	63	TTGACCC	60	CAGGTGT	23
AGCCCAC	43	GTTATTT	20	TACGTAC	63	TTGACCT	60	AGGGTGT	17
TACCCAC	42	CGTATTT	19	TTACGTC	60	GTGACCT	57	TGGGTGT	16
ATCCCAC	39	TCTATTT	19	CTACGTA	59	ATGACCT	45	GAGGTGC	15
TTCCCAC	27	ATTATTT	18	TACGTCA	53	CTGACCT	41	TCATCAC	13
AACCCAC	26	CCTGTTT	17	CGACGCA	51	TGACCCT	41	CTCACCT	12
GTCCCAC	23	TATATTT	17	TGACGTT	51	CTGACCC	31	CGGGTGT	12
Total	363	Total	173	Total	533	Total	456	Total	170

Foxj2		Foxp1		Junb		Sox14		Zscan20	

ATGTTTA	48	CGTAAAC	32	TGCCACA	12	CAATTCA	34	AGGGTTT	20
AACAAAC	44	GGTAAAC	30	AGAATTC	12	CAATTGA	30	AGGGTCG	16
GTGTTTA	41	AGTAAAC	28	AATCTTT	11	CAATAGT	26	AGGGTTG	14
AACAAAT	35	TGTAAAT	27	GTCAACA	10	CAATAAA	24	TACAGGT	14
TTGTTTT	35	AGTAAAT	23	ACGTTCC	8	CAATGTA	24	AGGGTCA	10
TTGTTTG	30	CGTAAAT	18	GTCCGTA	7	CAATAAC	22	AACTCTG	10
AACAAAA	28	ACTAAAC	17	TTGCGCT	7	CAATTAC	22	AGGGTCT	9
AACAAAG	28	CAACAGG	15	CGCCACA	7	CAATACT	21	GTAAGGT	9
TACAAAC	28	GGTAAAT	15	GCCGTAC	6	CAATACA	19	ACAATAG	8
Total	317	Total	205	Total	80	Total	222	Total	110

**Figure 5:**
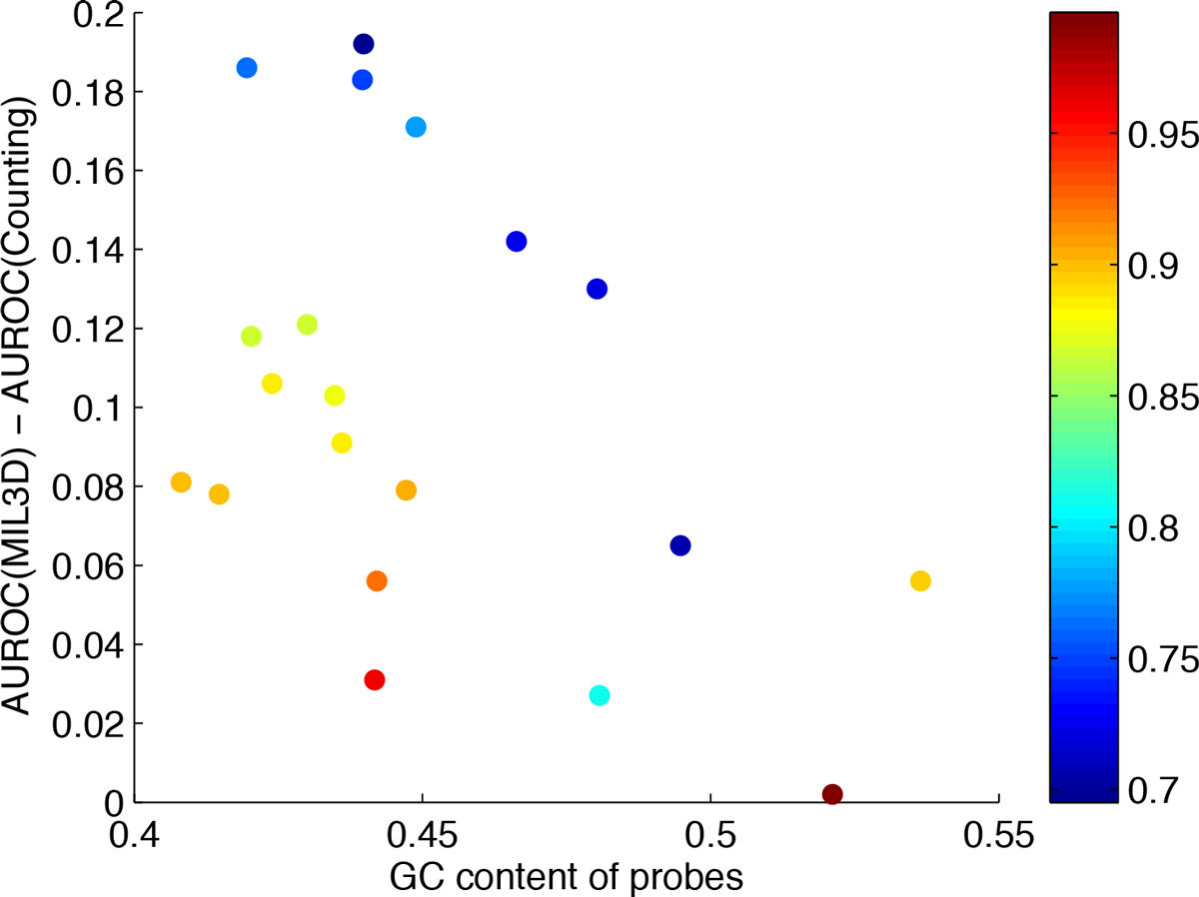
**Correlation between probe sequence GC content and performance gain by MIL3D**. A significant negative correlation (Pearson correlation coefficient = −0.44, *p*-value = 0.05) is observed between the GC content of the test probe sequence and the performance gain achieved by MIL3D. Color of data points represents the AUC by the simple counting method.

We also examined the correlation between the performance gain of MIL3D and the GC content of the probe sequences. It is well known that genomic sequences have non-uniform frequency of nucleotides and that certain promoter elements show strong preference for either the GC-rich or the GC-poor core promoters. While the collection of PBM probe sequences may not reflect the same GC content characteristics of real promoters, we observed an interesting, strong negative correlation between GC content of the probes and the performance gain by MIL3D (Figure 5, Pearson correlation coefficient = −0.44, *p*-value = 0.05); in contrast, the performance of 5-mer counting method is independent of GC content of the probe sequences (Pearson correlation coefficient = −0.03, *p*-value = 0.90). Vertebrate promoters are marked by an enrichment of both GC dinucleotide and G/C mononucleotide frequency. Therefore, the improved performance of our method in these relatively GC-poor regions may suggest that overall nucleotide composition rather than particular consensus pattern could have played a significant role in determining the binding affinity of the mouse TFs affected, which is also consistent with the results obtained above in correlation between performance and 7-mer occurrences. It will be interesting to investigate such relationships in multiple other organisms given that PBM data for those are now available, which may help understand TF-DNA interactions in the context of evolution.

## Conclusions

In this paper, we proposed a TFBS modeling and prediction approach - MIL3D. Combining MIL and structural properties of DNA, it models and predicts *in vitro *transcription factor bindings more accurately than the widely used *k*-mer counting methods on *in vitro *protein binding microarray data for twenty mouse transcription factors. Our analysis showed that the MIL3D approach can utilize subtle structural similarities when a strong sequence consensus is not available and demonstrated the encouraging potential of using MIL and structural properties of DNA to study molecular regulatory networks. The key idea in our method can be easily combined with current state-of-the-art *k*-mer based models, which often involve additional normalization, filtering, or transformation of data, to increase the prediction accuracy of TF-DNA interactions.

## Competing interests

The authors declare that they have no competing interests.

## Authors' contributions

ZG proposed the study, performed the data analysis and drafted the manuscript. JR conceived of the study, participated in its design and helped to draft the manuscript. All authors read and approved the final manuscript.

## Declarations

This article was funded by NSF grant IIS-1218201, NIH grants SC3GM086305, U54CA113001, G12MD007591, and a UTSA Tenure-track Research Award.

This article has been published as part of *BMC Genomics *Volume 16 Supplement 4, 2015: Selected articles from the IEEE International Workshop on Genomic Signal Processing and Statistics (GENSIPS) 2013. The full contents of the supplement are available online at http://www.biomedcentral.com/bmcgenomics/supplements/16/S4.
